# Editorial Change

**Published:** 1890-02

**Authors:** 


### Editorial Change.

The *Southern Dental Journal*, which has for eight years been in the editorial charge of Dr. B. H. Catching, Atlanta, Ga., has passed to other hands, and the doctor will have, for a time, rest from the responsibility of editorial work, and this he will no doubt enjoy. Dr. Catching is one of the independent and conscientious thinkers and writers of our profession; always ready to stand by his convictions.

The doctor will be missed from the editorial fraternity, but we will hope to hear from him often, for of course, after a little respite he will not be content to let his pen lie wholly idle, but will feel drawn, in some sort, to continue the communication he has so long had with the readers of the *Southern*. Of the new editor and new home of the *Southern Journal* we will be able to speak more definitely upon the receipt of the first number under the new management. We wish for it in the future, as in the past, abundant success.

